# How crystal structure prediction can impact small-molecule pharmaceutical development: past examples, success stories, and future prospects.

**DOI:** 10.1063/4.0000804

**Published:** 2025-10-27

**Authors:** Luca Iuzzolino

**Affiliations:** Merck & Co. Inc., Rahway, NJ, USA

## Abstract

The impact of crystal structure prediction on the development of small-molecule pharmaceutical products cannot be overstated. The choice of a thermodynamically metastable crystal structure as the lead development form, and the unexpected appearance of a lower energy phase, can have extremely detrimental consequences for patients, as well as negative financial and reputational impacts on pharmaceutical companies. Therefore, any technique that can minimize the risk of such an event occurring is extremely valuable. The power of computational crystal structure prediction comes from its ability to explore the crystal form landscape of a molecule without the limitations in terms of material availability, kinetic constraints, and characterization difficulties that experimental screens suffer from.

Therefore, they can predict the existence of more thermodynamically stable crystalline forms years before kinetic conditions lead to their appearance in real-life, which can lead to appropriate targeted experimental efforts. In this talk, some examples of crystal structure prediction, as well as some success stories, will be discussed. Potential areas of future developments, and some ongoing efforts to make them more practically useful, will also be highlighted.

